# Influences of Complexity on Decision Making in Young and Older Adults

**DOI:** 10.5964/ejop.v16i2.1958

**Published:** 2020-05-29

**Authors:** Stephen P. Badham, Calum A. Hamilton

**Affiliations:** a Department of Psychology, Nottingham Trent University, Nottingham, United Kingdom; b Institute of Neuroscience, Newcastle University, Newcastle, United Kingdom; Trinity College Dublin, Dublin, Ireland

**Keywords:** aging, decision-making, complexity, executive functioning, applied psychology

## Abstract

Leading theory hypothesizes that age deficits in decision making may rise as the complexity of decision-related information increases. This suggests that older adults would benefit relative to young adults from simplification of information used to inform decision making. Participants indicated political, nutritional and medical preferences and then chose between politicians, foods and medicines. The amount of information presented was systematically varied but age differences were largely similar for simple and complex trials. Paradoxically, the data showed that decisions based on simpler information could be less aligned with participant’s preferences than decisions based on more complex information. Further analyses suggested that participants may have been responding purely on the basis of their most valued preferences and that when information about those preferences was not presented, decision making became poorer. Contrary to our expectations, simplification of information by exclusion may therefore hinder decision making and may not particularly help older adults.

Higher life expectancy (Salomon et al., 2012) and the maturity of the WWII ‘baby boom’ generation (Van Bavel & Reher, 2013) has led to a large increase in the age of people in positions of power in business and politics (cf. Frey, Mata, & Hertwig, 2015). It is therefore becoming ever more important to understand age-related change and its impact on individuals and society, particularly because of the large range of cognitive differences shown between young and older adults (e.g., Craik & Salthouse, 2008; Deary et al., 2009). These factors have led to an exponential increase in cognitive aging research (Salthouse, 2010), including research into the role of aging in decision making (Strough, Löckenhoff, & Hess, 2015). The process of making decisions is crucial to everyday functioning such as deciding who to vote for, which foods are best to eat and what medicine to take. However, there has been less cognitive research evaluating older adults’ decision making in these applied contexts and this has been identified as an area in particular need of further research (Carstensen & Hartel, 2006).

Aspects of executive functioning/cognitive control that are involved in decision making have been shown to decline in healthy older adults. Decision making benefits from increased working memory (Hinson, Jameson, & Whitney, 2003) and inhibitory control (Del Missier, Mäntylä, & Bruine de Bruin, 2010) and leading theories of age-related cognitive decline propose age deficits in working memory (e.g. Craik, 2000; Craik & Byrd, 1982) and inhibition (e.g., Hasher & Zacks, 1988) as well as executive functioning in general (e.g., Allain et al., 2007; Buckner, 2004; West, 1996). Broadly, the role of executive functioning in decision making (e.g., Del Missier, Mäntylä, & Bruine de Bruin, 2012) has been used to explain many age-related deficits that are found in decision-making literature (see Brand & Markowitsch, 2010; Denburg & Hedgcock, 2015, for reviews).

The influence of task complexity has been linked to executive functioning in age comparisons of decision making, usually showing age deficits for tasks of increasing complexity (e.g., see Brand & Markowitsch, 2010; Peters & Bruine de Bruin, 2012, for reviews). For example, during complex decision making, older adults (relative to young adults) can show difficulty applying rules and greater susceptibility to the way decision-related material is framed/presented (Bruine de Bruin, Parker, & Fischhoff, 2007). Similarly, Finucane et al. (2002) and Finucane, Mertz, Slovic, and Schmidt (2005) showed that when information needed to be processed before decisions could be made (e.g., combinations of percentages weighed against one another), age deficits were particularily large and they also showed greater inconsistency in older adults’ decisions relative to young adults’ decisions when the same information was presented in different ways (e.g., ordered vs. unordered).

Most relevant to the current study are age differences in decision strategies that investigate complexity due to the *amount* of information utilized by young and older adults. Besedeš, Deck, Sarangi, and Shor (2012) showed that decisions involving more information led to greater age deficits than decisions involving less information. A variety of studies on information seeking show that older adults seek less information than do young adults, indicating that they do this in order to minimize the use of executive functioning (Mather, 2006). In a decision-making simulation, management teams of older adults engaged in less information searching than management teams of young adults (Streufert, Pogash, Piasecki, & Post, 1990), older women have been shown to seek less information than young women when making medical-treatment decisions about breast cancer (Meyer, Russo, & Talbot, 1995), and in a more abstract task involving evaluation of diamonds, older adults sought less information than did young adults (Mata, Schooler, & Rieskamp, 2007). A review by Liu, Wood, and Hanoch (2015) also indicated that older adults prefer making decisions with fewer options. In contrast to the above, some research has shown similar age deficits in decisions based on more or less information (Finucane et al., 2005) and similar information seeking in young and older adults (e.g., Queen, Hess, Ennis, Dowd, & Grühn, 2012). However, overall the literature suggests that older adults improve relative to young adults when decisions are simpler.

In the current study, we aimed to evaluate if young and older adults could make better decisions when less information was presented by manipulating the amount of information available for decision making across simple and complex trials. We are only aware of five aging studies with such a manipulation, three which showed that age deficits increased with complexity (larger age deficits for more complex trials, Besedeš et al., 2012; Frey et al., 2015; Hanoch et al., 2011) and two that did not (Finucane et al., 2005; Queen et al., 2012). Despite the mixed results, the theoretical consensus reviewed above seems to be that older adults should perform disproportionally worse as decision complexity increases. Peters and Bruine de Bruin (2012) argued that policy makers and practitioners seeking to inform older adults’ decisions should provide simpler materials that are summarized into fewer key points. This is an important issue to resolve as it is essential that older adults are provided with the best opportunity to make optimum decisions.

In previous studies that manipulated complexity, simpler trials often involved a reasonably large amount of information. Besedeš et al. (2012) had four options, each with multiple attributes and Finucane et al. (2005) and Hanoch et al. (2011) had at least three options, each with multiple attributes. All of these studies involved decisions based on new information learned in the experiment that may have placed larger demands on working memory. Even the least difficult measure of working memory span (forward digit span) in a meta-analysis by Bopp and Verhaeghen (2005) shows mean span scores of seven items for older adults. This suggests that older adults’ working memory capacity may have been exceeded, even in simple trials. The study by Queen et al. (2012) had participants base their decisions on their own preferences which would have reduced memory requirement, nonetheless their simpler trials still involved five options, each with five attributes and the attributes were only visible one at a time (requiring additional mental retention). Finally, the study by Frey et al. (2015) had just two options in their simple trials but participants made their decisions after sampling multiple pieces of numerical information which was allowed to vary between young and older adults (their study was more focused on strategy use than complexity). In the current study, decisions involved just two options with simple trials consisting of one to three attributes based on participants’ real-life preferences. Overall, the current study provided the optimal conditions for older adults to perform well in simple trials, therefore evaluating the potential for information simplification to reduce age deficits in decision making.

## Method

### Design

Young and older participants filled in basic data about their political, nutritional and medical preferences in three conditions. Following each of these, they made a series of binary decisions about politicians, food and medicines. For each condition there were simple decisions based on minimal information and complex decisions based on multiple pieces of information. The overall design was age (young, older; between participants) x topic^i^ (political, nutritional and medical; within participants) x complexity (simple, complex; within participants).

### Participants

Thirty young adults (22 female) aged 19–30 years (*M* = 24.4, *SD* = 3.3) and 30 healthy older adults (19 female) aged 61–82 years (*M* = 71.7, *SD* = 4.5) took part in the experiment. Young participants were recruited from Nottingham Trent University and reported no issues with eyesight, hearing or reading. Older participants were recruited from the university’s Trent Aging Panel which is populated by the local community; their self-rated corrected eyesight, hearing, and general health averaged 3.8, 4.0, and 3.9 (equivalent to “good”), respectively, on a five-point scale (1 = “very poor” to 5 = “very good”). All participants provided written informed consent and the study was approved by Nottingham Trent University’s research ethics committee.

To assess cognitive functioning, participants completed the Digit Symbol Substitution test from the Wechsler Adult Intelligence Scale – Revised (Wechsler, 1981) as a measure of processing speed and the multiple choice part of the Mill Hill vocabulary test (Raven, Raven, & Court, 1988) as a measure of crystallized intelligence. The results were consistent with the literature (e.g., Salthouse, 2010); young adults performed better than older adults at the speed task, *t*(58) = 9.44, *p* < .001, *d* = 2.44 (*M*_young_ = 74.33, *SD*_young_ = 9.25; *M*_older_ = 49.47, *SD*_older_ = 11.07) and older adults performed better than young adults at the vocabulary task, *t*(58) = 5.89, *p* < .001, *d* = 1.52 (*M*_young_ = 18.90, *SD*_young_ = 2.91; *M*_older_ = 23.57, *SD*_older_ = 3.22).

### Materials and Procedure

All materials were presented digitally on a desktop PC running OpenSesame software (Mathôt, Schreij, & Theeuwes, 2012). The three conditions within the topic factor (political, nutritional and medical) were presented in blocks and were fully counterbalanced in their order of presentation. Participants were allowed to rest between each task and condition.

#### Political Condition

Initially, participants were asked how they would assign £100 million of government spending between education, healthcare and defense. Three boxes were provided and participants typed in three values (in millions) that added up to 100. Assigning exactly 1/3 to each sector was prohibited to aid later analysis (see Table 1 for means, which show largely similar preferences for young and older adults).

**Table 1:** Mean Initial Preferences for Young and Older Adults for Each Experimental Condition

Age Group	*M*	*SD*
Political Education Spending		
Young	37.73	7.263
Older	34.83	5.983
Health Spending		
Young	42.73	10.017
Older	41.03	7.384
Defence Spending		
Young	19.53	10.471
Older	24.13	8.513
Nutritional Energy Rank		
Young	2.23	1.612
Older	2.87	1.502
Total Fat Rank		
Young	2.70	1.489
Older	3.43	1.612
Carbohydrates Rank		
Young	3.97	1.402
Older	3.53	1.432
Fibre Rank^a^		
Young	4.73	1.388
Older	3.83	1.392
Protein Rank		
Young	3.10	1.470
Older	2.87	1.737
Salt Rank		
Young	4.27	1.437
Older	4.47	1.943
Medical Joint/Muscle Pain Unpleasantness		
Young	5.03	2.092
Older	5.03	2.236
Nausea Unpleasantness		
Young	7.20	1.540
Older	6.23	2.388
Headache Unpleasantness^a^		
Young	6.07	1.982
Older	5.00	1.912
Drowsiness Unpleasantness		
Young	3.53	2.177
Older	3.47	2.636

^a^Age difference significant at *p* < .05.

**Figure 1:**
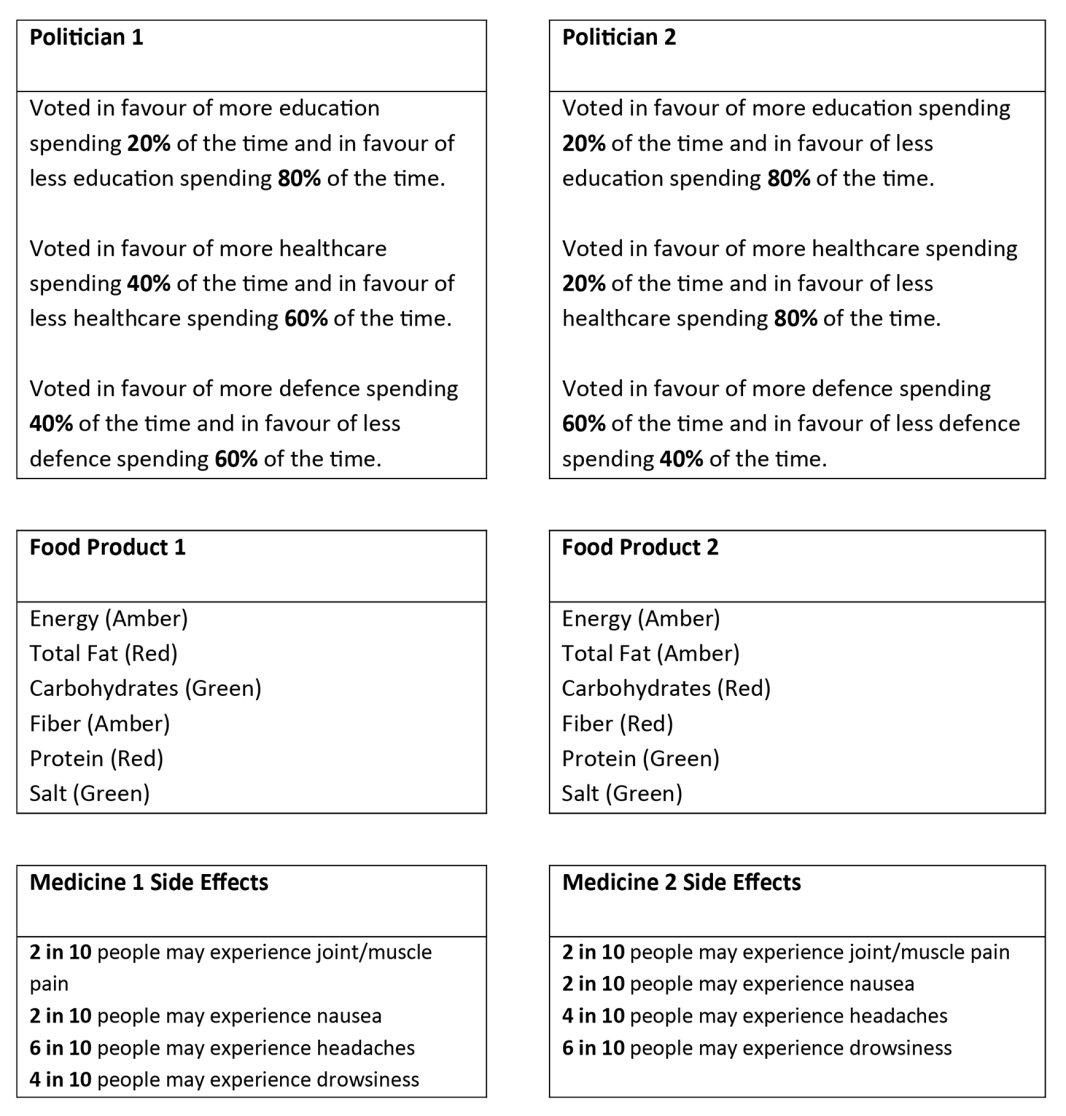
Example of a complex trial in the political condition (top), the nutritional condition (middle) and the medical condition (bottom). Simple trials were presented identically but with items removed from each option. The participant must press 1 or 2 on the keyboard to indicate their choice of the left or right option. The traffic-light colors in parenthesis for the nutritional condition are displayed in text here but were the font colors in the original experiment.

Following the assignments, participants were asked to choose between two politicians. In the complex trials, education, health and defense voting choices were indicated for each politician (see Figure 1).In the simple trials, information about just one spending sector (e.g., just education voting) was provided. Thirty complex trials and 18 simple trials were constructed. Only percentages of 20, 40 and 60 were used to indicate the proportion of votes each politician made in favor of more or less spending in a given sector. For complex trials, the voting behavior always added up so that a given politician was voting for more spending exactly 1/3 of the time (i.e., the three percentages indicated for more spending always added up to 100 and the three percentages indicated for less spending always added up to 200; see Figure 1). The above constraints meant that there were six ways to assign a given voting profile for a politician. This meant that there were five ways of assigning a different profile for the opposite politician. Therefore 6 x 5 = 30 complex trials were presented. For simple trials, there were only six ways to assign different percentages to each politician and this was done for each of the three spending sectors, resulting in 18 simple trials. During the experiment, complex and simple trials were presented randomly, intermixed in a single block. At the end of the block, participants were asked again to assign their £100 million spending budget. This was to later check that participants had not forgotten their initial assignments.

#### Nutritional Condition

In this condition participants initially ranked how important the following six nutritional properties of food were to them personally: Energy, Total Fat, Carbohydrates, Fibre, Protein and Salt. The factors were listed on the screen and participants ranked them from one to six with one being the most important to them and six being the least important to them (see Table 1 for means, which show largely similar preferences for young and older adults). This was done by using the number keys after clicking a box next to each property. All ranks from one to six had to be used once and only once.^ii^

Following the rankings, participants were asked to choose between two foods ‘*which tasted and looked the same* but had different nutritional properties’. The nutritional properties were listed in two grids (see Figure 1) and a traffic-light system was used to indicate if a property was good (green font), average (amber font) or bad (red font). This was done to avoid using specific measurements of quantity that participants may not be familiar with. Additionally, if one participant thought more fat was good and another thought less fat was good, the traffic light system would cause them to both respond in the same way as long as they both viewed fat with similar levels of importance based on rank.

In the complex trials, information about all six nutritional properties was presented for each food. There were always two good, two average and two bad properties for each food. Forty-eight different combinations were created and a random subset of 24 of these was selected separately for each participant. In the simple trials, information about three nutritional properties was presented for each food (the same three properties were always used for both foods in a given trial). There was always one good, one average and one bad property for each food. Again, 48 different combinations were created and a random subset of 24 of these was selected separately for each participant. During the experiment, complex and simple trials were presented randomly, intermixed in a single block. At the end of the block, participants ranked the nutritional properties again.

#### Medical Condition

In this condition, participants initially rated their opinion about four medicinal side effects: joint/muscle pain, nausea, headaches, and drowsiness. They were presented with the four side effects and rated each of them on a 10- point Likert scale from 1 slightly unpleasant to 10 extremely unpleasant (see Table 1 for means, which show largely similar preferences for young and older adults).

Following the ratings, participants were asked to choose between two ‘*equally effective* medicines’ based on their side effects. The side effects for the medicines were presented in two grids (see Figure 1). Each side effect was listed as the number of people out of 10 who experience this side effect (e.g., ‘2 in 10 people may experience nausea’). In the complex trials, all four side effects were presented for each medicine. In the simple trials, two side effects were presented for each medicine and these were always the same two types of side effect for both medicines.

For the complex trials, the numbers out of 10 for the four side effects were always a 2, another 2, a 4 and a 6 (therefore, the sum frequency of the combined four side effects was always 14 out of 40). For a given medicine this resulted in 12 possible combinations across the four side effects leaving 11 possible different combinations for the other medicine in the same trial. This resulted in 12 x 11 = 132 possible complex trials and a random subset of 24 of these was selected separately for each participant. For the simple trials, the numbers out of 10 for the two side effects presented always summed to eight (i.e., either a 2 and a 6, or a 4 and another 4). This resulted in six possible combinations of different side-effect frequencies across the two medicines and there were also six ways to choose two out of the four side effects to present. Therefore, 6 x 6 = 36 different simple trials were possible and a random subset of 24 of these was selected separately for each participant. During the experiment, complex and simple trials were presented randomly, intermixed in a single block. At the end of the block, participants rated the unpleasantness of the side effects again.

## Results

### Scoring

#### Political Condition

The proportion of money that the participants assigned to each spending sector was compared to the proportion of the time each politician voted in favor of more spending in that sector. Given that in the complex trials the politicians always voted for more spending exactly 1/3 of the time, the proportion of their spending in each sector was comparable to the proportion of money the participant would assign to that sector. The root mean square error (RMSE) was calculated between the participant’s spending habits and each of the two politicians’ spending habits for each trial. Decision accuracy for each trial was determined by establishing if the participant chose the politician with the smaller RMSE in relation to the participant’s spending. Trials where RMSE was equal for the two politicians were excluded from the accuracy measurement (5% of complex and 17% of simple trials). The overall accuracy proportion and the mean RMSE between the participant’s spending and their chosen politicians were dependent variables. These values were calculated separately for the participant’s preferences at the start and end of the block. This was done to confirm that participants were not forgetting their initial choices which may have had a greater impact on older adults who typically have poorer memory performance than do young adults (Naveh-Benjamin & Ohta, 2012). Additionally, a separate RMSE difference between the reported start and end preferences was calculated for each participant.

#### Nutritional Condition

The ranks of the six nutritional properties chosen by the participants were used to compute a score for each food in each trial. Firstly, the participants’ chosen ranks were inverted so that 6 was the most important and 1 was the least important. Then for each food, good properties were given a score of +1, average properties a score of 0 and bad properties a score of -1. A score for each nutritional property was computed by multiplying the participant’s inverted rank by the score for that property. Then a score for each food was computed by summing the scores for each property.^iii^ Therefore, properties considered more important by the participant would have a greater influence on the overall score for a given food and higher scores would correspond to a more desirable food. The food scores were used to compute the optimal (accurate) choice for a participant on a given trial. Similar to the political condition, the overall accuracy and mean scores for the chosen food were computed as dependent variables separately for the nutritional property ranks indicated by the participant at the start of the block and for the nutritional property ranks indicated by the participant at the end of the block (a separate RMSE difference between the raw start and end ranks was also calculated for each participant). Trials resulting in identical scores for each food were excluded from the accuracy measurement (6% of complex and 6% of simple trials had no correct response and were excluded from the accuracy analysis).

#### Medical Condition

The ratings for the unpleasantness of each side effect indicated by the participants were used to establish the unpleasantness of each medicine in a trial. Firstly, the participants’ ratings were weighted so that they summed to one. (e.g., if a participant rated joint/muscle pain, nausea headaches, and drowsiness as 1, 2, 9 and 10 respectively then each value would be divided by the overall sum to give 0.05, 0.09, 0.41, and 0.45). Likewise, for each medicine, the frequency of occurrence of each side effect was weighted so that the sum of all the frequencies was 1 (e.g., 1/10, 2/10, 4/10 and 5/10 would be 0.08, 0.16, 0.33 and 0.42, respectively). This allowed comparison between a participant’s preferences and a medicine to be made across a fixed range of 0-1. The RMSE was then calculated between the weighted participants’ ratings and the weighted frequencies of occurrence of each side effect for each medicine for each trial. An accurate response was established by determining if the participant chose the medicine with the *maximum* RMSE compared to their preferences (note that we chose the maximum RMSE because participants were trying to avoid unpleasant side effects, unlike the political condition where participants tried to choose a politician with similar views). For the complex trials, the weights were based on all four ratings; for the simple trials, the weights were based on the two ratings relevant to side effects presented (e.g., if just nausea and headaches were relevant with ratings of 2 and 9, respectively, then their weights would be 0.18 and 0.82, respectively). Again, similar to the political and nutritional conditions, overall accuracy and mean RMSE between the participants rating and their chosen medicine for each trial were calculated separately for ratings made at the start and end of the block (a separate RMSE difference between the raw start and end ratings was also calculated for each participant). Trials resulting in identical RMSE values for both medicines were excluded from the accuracy measurement (8% of complex and 5% of simple trials had no correct response and were excluded from the accuracy analysis).

### Analysis

Throughout the article, standard null hypothesis tests are accompanied by an estimated Bayes Factor implemented through JASP computer software (Love et al., 2015). The Bayes Factor (*BF*_10_) provides an odds ratio for the alternative/null hypotheses (values < 1 favor the null hypothesis and values > 1 favor the alternative hypothesis). For example, a *BF*_10_ of 0.40 would indicate that the null hypothesis is 2.5 times more likely than the alternative hypothesis (see Jarosz & Wiley, 2014). All Bayesian *t*-tests are two-tailed using the standard Cauchy prior width of 0.707.

#### Overall Analysis

**Figure 2:**
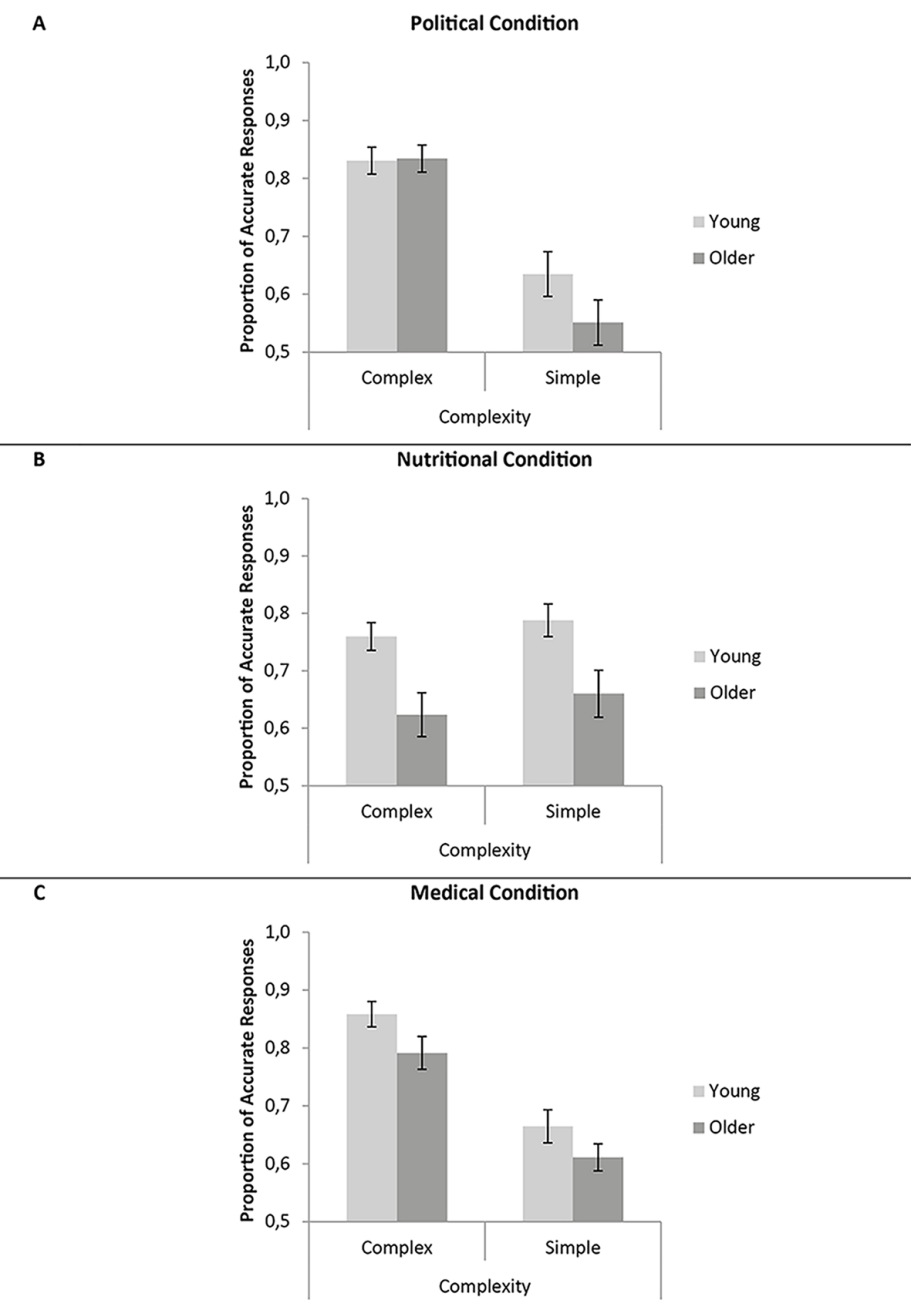
Proportion of accurate responses (.5 = chance) for young and older adults when making complex and simple decisions based on their political (A) nutritional (B) and medical (C) preferences. Error bars are ± 1*SE*.

The only comparable measure across the topic factor was the accuracy of each decision with respect to each participants’ stated preferences. This was assessed in a 2 (age; young, older) x 3 (topic; political, nutritional, medical) x 2 (complexity; complex, simple) mixed ANOVA (see Figure 2, for means) where accuracy was determined by the preferences indicated by participants at the start of a block.^iv^ Young adults made more accurate choices based on their preferences than did older adults, *F*(1, 55) = 9.98, *MSE* = 0.050, *p* = .003, $\eta_{p}^{2}$ = .154, *BF*_10_ = 11.172. Participants responded with similar decision accuracy across the three topics for the standard, *F* < 1, but not for the Bayesian, *BF*_10_ = 1.579 x 10^7^, analyses: performance was highest in the medical condition then the political condition and then the nutritional condition. Surprisingly, participants were more accurate for complex trials involving more information in each decision than for simple trials involving less information in each decision, *F*(1, 55) = 94.55, *MSE* = 0.016, *p* < .001, $\eta_{p}^{2}$ = .632, *BF*_10_ > 10^15^. Age did not interact with complexity, *F* < 1, *BF*_10_ = 0.381, or with topic, *F*(2, 110) = 2.12, *MSE* = 0.038, *p* = .125, $\eta_{p}^{2}$ = .037, *BF*_10_ = 3.767, and there was no triple interaction between age, topic and complexity, *F*(2, 110) = 2.17, *MSE* = 0.011, *p* = .119, $\eta_{p}^{2}$ = .038, *BF*_10_ = 0.321. There was an interaction between topic and complexity, *F*(2, 110) = 53.43, *MSE* = 0.011, *p* < .001, $\eta_{p}^{2}$ = .493, *BF*_10_ = 8.721 x 10^7^, which is explored below. The same patterns of significance were found when basing accuracy from the participants’ preferences given at the end of each block, indicating that the observed patterns were not due to participants forgetting their reported preferences.

#### Political Condition

A 2 (age; young, older) x 2 (complexity; complex, simple) mixed ANOVA was conducted on the accuracy of political decisions based on participants’ preferences indicated at the start of a block. There was no main effect of age, *F*(1, 58) = 1.14, *MSE* = 0.042, *p* = .291, $\eta_{p}^{2}$ = .019, *BF*_10_ = 0.550. Participants were more accurate for complex trials than for simple trials, *F*(1, 58) = 90.39, *MSE* = 0.019, *p* < .001, $\eta_{p}^{2}$ = .609, *BF*_10_ = 2.806 x 10^11^. There was no interaction between age and complexity, *F*(1, 58) = 3.00, *MSE* = 0.019, *p* = .088, $\eta_{p}^{2}$ = .049, *BF*_10_ = 1.077. The actual RMSE data were also analyzed but because RMSE was calculated differently for complex and for simple trials, only the effects involving age were meaningful to report. *T*-tests showed that older adults performed *better* than young adults (lower RMSE) for complex trials, *t*(50.27) = 2.28, *p* = .027, *d* = 0.59, *BF*_10_ = 2.227, (*M*_young_ = 14.43, *SD*_youn_*_g_* = 2.91; *M*_older_ = 12.97, *SD*_older_ = 1.92), and there were no age deficits for simple trials, *t* < 1, *d* = 0.15, *BF*_10_ = 0.303, (*M*_young_ = 15.05, *SD*_young_ = 2.99; *M*_older_ = 15.52, *SD*_older_ *=* 3.32). These results may differ from the accuracy data because trials were excluded from the accuracy data when there was no correct choice but the RMSE data include all trials and the RMSE was calculated for whichever option was chosen, regardless of whether or not it was correct. Additionally, there was no evidence that older adults were changing their mind about their political preferences throughout the experiment due to age-related memory impairment: The difference between a participant’s preferences at the beginning of the political block and their preferences at the end of the political block (also based on RMSE, lower is better) was calculated and compared for young and older adults and there was no significant effect (*t* < 1, *d* = 0.07, *BF*_10_ = 0.270, *M*_young_ = 1.75, *SD*_young_ = 4.06; *M*_older_ = 1.49, *SD*_older_ = 3.35).

The fact that participants performed better for complex trials than for simple trials is interesting because we would naturally predict the opposite. However, there is a factor that could influence participants’ behavior for simple trials, namely that the two politicians to choose from were not equated in their spending voting as they were for complex trials. In complex trials, both politicians always voted for more spending exactly 1/3 of the time so more spending in one sector can be cancelled out by less spending in another. For simple trials, where each politician’s spending for only one sector is presented, there is always one politician who votes for more public spending than the other. The data were analyzed to see how often a given participant chose the more generous (higher spending voting) politician for simple trials. One-sample *t*-tests showed that both young and older adults selected the more generous politician more than half the time (*t*_young_(29) = 7.80, *p* < .001, *d* = 5.23, *BF*_10_ = 9.971 x 10^5^, *M*_young_ = 0.68, *SD*_young_ = 0.13; *t*_older_(29) = 8.29, *p* < .001, *d* = 4.63, *BF*_10_ = 3.156 x 10^6^, *M*_older_ = 0.74, *SD*_older_ = 0.16). There was also no significant age difference in the choice of the more generous politician, *t*(58) = 1.46, *p* = .151, *d* = 0.38, *BF*_10_ = 0.636. Therefore the participants have a natural bias that hinders performance for simple trials in the political condition so this could explain the superior performance in complex trials. However, this problem does not occur for the nutritional and medical conditions where the simple trials have more than one item which allows the two options to be balanced.

#### Nutritional Condition

A 2 (age; young, older) x 2 (complexity; complex, simple) mixed ANOVA was conducted on the accuracy of nutritional decisions based on participants’ preferences indicated at the start of a block. Young adults were more accurate than were older adults, *F*(1, 55) = 9.03, *MSE* = 0.055, *p* = .004, $\eta_{p}^{2}$ = .141, *BF*_10_ = 7.119. There was no effect of complexity, *F*(1, 55) = 3.45, *MSE* = 0.009, *p* = .072, $\eta_{p}^{2}$= .059, *BF*_10_ = 0.706, although the numerical trend was in the predicted direction with better performance for simple trials than for complex trials. There was no interaction between age and complexity, *F* < 1, *BF*_10_ = 0.466. The scores for each food chosen by a participant (see above for scoring system; higher is better) based on their preferences at the start of a block were analyzed but due to the fact that scores were calculated differently for simple and complex trials, only effects involving age were meaningful to report. *T*-tests showed that older adults performed worse than young adults for complex trials, *t*(55) = 3.74, *p* < .001, *d* = 0.99, *BF*_10_ = 61.035 (*M*_young_ = 1.70, *SD*_young_ = 0.77; *M*_older_ = 0.69, *SD*_older_ = 1.21), but not for simple trials, *t*(48.51) = 1.42, *p* = .163, *d* = 0.38, *BF*_10_ = 0.622 (*M*_young_ = 0.85, *SD*_young_ = 0.73; *M*_older_ = 0.51, *SD*_older_ = 1.03). The difference between participants’ ranks at the start of the nutrition block and their ranks at the end of the nutrition block (based on RMSE, lower is better) were calculated and compared for young and older adults. Young adults were more consistent than were older adults, *t*(55) = 2.15, *p* = .035, *d* = 0.57, *BF*_10_ = 1.795, (*M*_young_ = 0.58, *SD*_young_ = 0.59; *M*_older_ = 0.99, *SD*_older_ = 0.81). Therefore, the above analyses were repeated using accuracy and RMSE values based on the participants’ ratings provided at the end of the block and there were no changes in significance to those reported above. Overall, the nutritional condition behaves more like our predictions; complex trials were (numerically) more difficult than simple trials, age deficits were present and these deficits were more evident for the complex trials compared to the simple trials.

#### Medical Condition

A 2 (age; young, older) x 2 (complexity; complex, simple) mixed ANOVA was conducted on the accuracy of medical decisions based on participants’ preferences indicated at the start of a block. Older adults were marginally less accurate than were young adults, *F*(1, 58) = 3.76, *MSE* = 0.029, *p* = .057, $\eta_{p}^{2}$ = .061, *BF*_10_ = 1.170, accuracy was better for complex trials compared to simple trials, *F*(1, 58) = 99.38, *MSE* = 0.011, *p* < .001, $\eta_{p}^{2}$ = .631, *BF*_10_ = 1.076 x 10^12^, and there was no interaction between age and complexity, *F* < 1, *BF*_10_ = 0.594. The RMSE data were also analyzed based on participants’ ratings of side effects indicated at the start of the medical block. Here participants were trying to avoid choosing a medicine which had side effects they did not like so higher RMSE between their ratings and their chosen medicine’s side effects reflects a better choice. The RMSE was calculated differently for complex and simple trials so only effects involving age were meaningful to report. *T*-tests showed that there was no age difference for complex trials, *t* < 1, *d* = 0.00, *BF*_10_ = 0.263 (*M*_young_ = 0.16, *SD*_young_ = 0.02; *M*_older_ = 0.16, *SD*_older_ = 0.03), and no age difference for simple trials, *t* < 1, *d* = 0.18, *BF*_10_ = 0.311 (*M*_young_ = 0.25, *SD*_young_ = 0.05; *M*_older_ = 0.24, *SD*_older_ = 0.06). The difference between participants’ ratings at the start of the medical block and their ratings at the end of the medical block (based on RMSE, lower is better) were calculated and compared for young and older adults. Young adults were more consistent than were older adults, *t*(58) = 2.27, *p* = .027, *d* = 0.58, *BF*_10_ = 2.179, (*M*_young_ = 0.71, *SD*_young_ = 0.58; *M*_older_ = 1.17, *SD*_older_ = 0.95). Therefore, the above analyses were repeated using accuracy and RMSE values based on the participants’ ratings provided at the end of the block and there were no changes in significance to those reported above. Overall, there were no interesting effects of age in the medical condition. More interesting is the fact that participants were able to perform better for complex trials compared to simple trials. Unlike the political condition, there was no systematic bias in the simple trials that could have explained poor performance because the overall frequency of side effects was the same for both choices in simple trials.

#### Single-Dimension Analysis

The higher performance for complex trials than for simple trials for the political and medical conditions was unexpected so further analyses were conducted based on whichever aspect of the participant’s preferences was most dominant. If, for example, a participant in the political condition cared much more about education than healthcare and defense, then they may have chosen their politician purely on the basis of education. This would lower their performance for some of the simple trials where education was not presented. The accuracy scores were recomputed assuming that participants were trying to respond on the basis of their most extreme preference, ignoring other items. All trials where a single-dimensional response could not be determined were excluded. This occurred when the maximum preference was not presented in simple trials (24.5% of overall trials) or when a participant had multiple maximum preferences with contradicting ideal responses (7.5% of overall trials).

**Figure 3:**
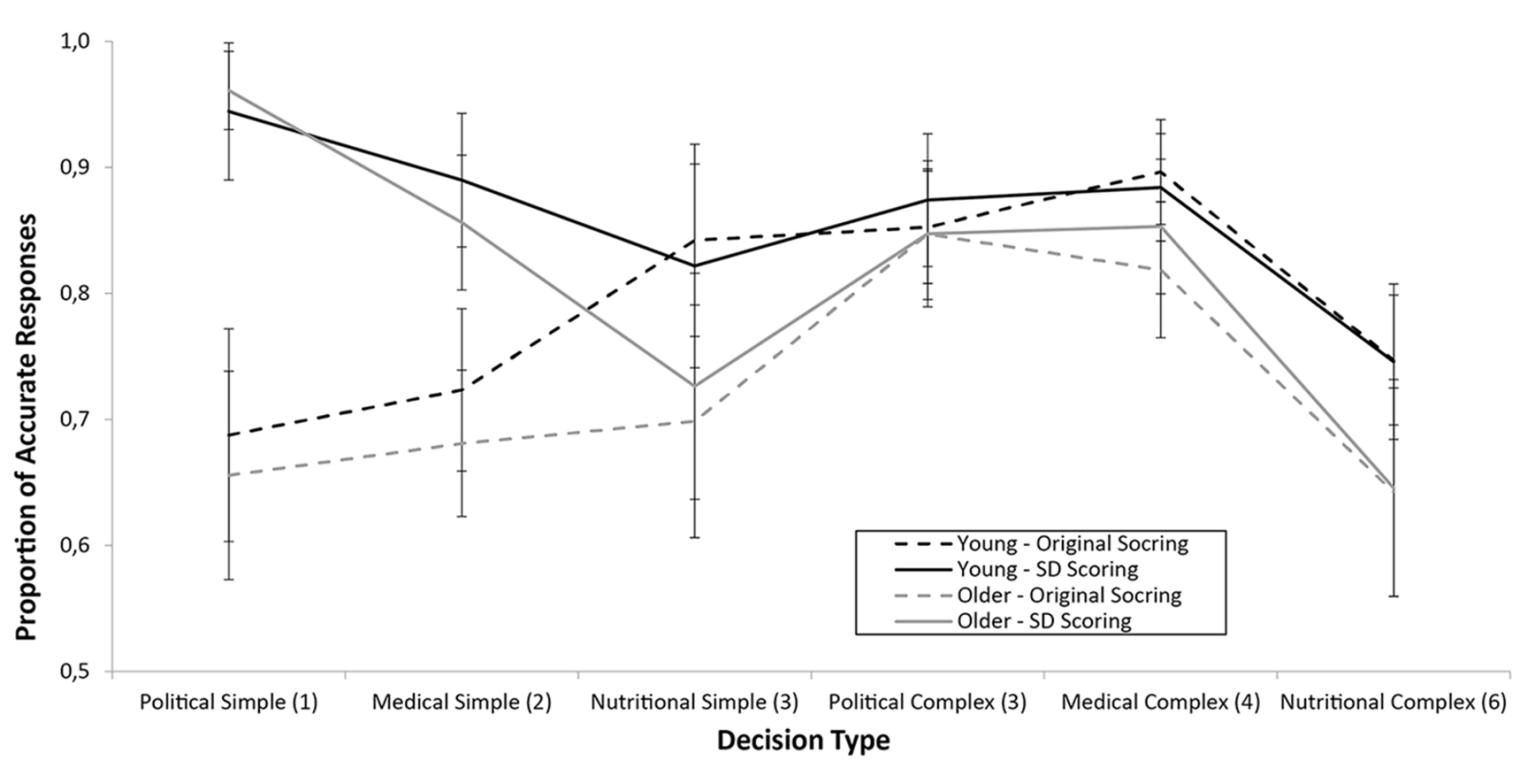
Proportion of accurate responses (.5 = chance) for young and older adults based on original scoring or single-dimensional (*SD*) scoring. The x-axis shows the decision types in order of the number of attributes (indicated in parentheses) involved in each decision. Error bars are ± 95% CI.

A 2 (age; young, older) x 3 (topic; political, nutritional, medical) x 2 (complexity; complex, simple) x 2 (Accuracy measure; original measures outlined above, single dimensional) mixed ANOVA was conducted on the accuracy data (based on preferences indicated at the start of each condition) for only trials where a single dimensional measure of accuracy could be ascertained (i.e., regardless of accuracy measure, the exact same trials were used). Figure 3 shows the means. Violations of sphericity were accounted for by applying Greenhouse-Geisser corrections to degrees of freedom. Young adults responded more accurately than did older adults, *F*(1, 55) = 5.60, *MSE* = 0.089, *p* = .021, $\eta_{p}^{2}$ = .092, *BF*_10_ = 3.371. Accuracy varied across topic; political > medical > nutritional, *F*(1.70, 93.53) = 9.19, *MSE* = 0.095, *p* < .001, $\eta_{p}^{2}$ = .143, *BF*_10_ > 10^15^. There was no effect of complexity with standard statistics, *F*(1, 55) = 1.053, *MSE* = 0.025, *p* = .309, $\eta_{p}^{2}$ = .019, but there was with Bayesian statistics, *BF*_10_ = 2.654 x 10^9^. Interestingly, accuracy was higher when determined via a single dimensional measure as opposed to the original RMS/scoring measures described above, *F*(1, 55) = 63.10, *MSE* = 0.017, *p* < .001, $\eta_{p}^{2}$ = .534, *BF*_10_ > 10^15^, indicating that participants were responding mainly on the basis of their maximum preferences. There were interactions between topic and accuracy measure, *F*(2, 110) = 21.39, *MSE* = 0.013, *p* < .001, $\eta_{p}^{2}$ = .280, *BF*_10_ = 8.516 x 10^4^, between complexity and accuracy measure, *F*(1, 55) = 99.92, *MSE* = 0.008, *p* < .001, $\eta_{p}^{2}$ = .645, *BF*_10_ = 3.128 x 10^7^, and a triple interaction between topic, complexity and accuracy measure, *F*(2, 110) = 31.26, *MSE* = 0.008, *p* < .001, $\eta_{p}^{2}$ = .362, *BF*_10_ = 1211: Figure 3 shows that the single-dimensional measures are at least as accurate as the original scoring measures, particularly for decisions involving fewer items. There was also an interaction between topic and complexity, *F*(2, 110) = 17.50, *MSE* = 0.020, *p* < .001, $\eta_{p}^{2}$ = .241, *BF*_10_ = 1.059 x 10^6^, revealing a qualitatively similar pattern to the earlier data depicted in Figure 2 (but attenuated by the single-dimensional measure where simple trials were more accurate than complex trials). Finally, there were no interactions involving age, indicating that both age groups were responding similarly to the task (*F*s < 2.06; Age x Topic *BF*_10_ = 7.246, Age x Complexity *BF*_10_ = 0.124, Age x Accuracy Measure *BF*_10_ = 0.190, Age x Topic x Complexity *BF*_10_ = 0.056, Age x Topic x Accuracy Measure *BF*_10_ = 0.069, Age x Complexity x Accuracy Measure *BF*_10_ = 0.023, Age x Topic x Complexity x Accuracy Measure *BF*_10_ = 2.759 x 10^-4^). Table 2 shows the number of young and older adults performing more accurately with the original or single dimensional measures for each type of decision (participants with equal accuracy for both measures are excluded). A further ANOVA was conducted using just the single-dimensional measure, retaining the age, topic and complexity factors outlined above. Notably, there was no longer a main effect of age, *F*(1, 55) = 2.78, *MSE* = 0.055, *p* = .101, $\eta_{p}^{2}$ = .048, *BF*_10_ = 1.364, and performance on simple trials now exceeded that of complex trials, *F*(1, 55) = 21.94, *MSE* = 0.013, *p* < .001, $\eta_{p}^{2}$ = .048, *BF*_10_ = 1021.

**Table 2:** Number of Young and Older Adults Showing Higher Accuracy With Original or Single-Dimensional (SD) Scoring for Each Decision Type

Age Group and Measure	Political Simple (1)	Medical Simple (2)	Nutritional Simple (3)	Political Complex (3)	Medical Complex (4)	Nutritional Complex (6)
Young Original Scoring	1	1	11	6	10	14
Young *SD* Scoring	19	24	6	13	8	14
Older Original Scoring	0	1	7	12	3	13
Older *SD* Scoring	22	25	12	12	9	12

*Note.* Numbers in parentheses show the number of attributes involved in each decision.

## Discussion

The data showed that manipulating the amount of information involved in political, nutritional and medical decisions can have unpredictable effects and that policy makers and practitioners should be cautious when simplifying information that needs to be conveyed to aid decision making. Our main analyses showed that excluding information in order to simplify decisions sometimes led to a *reduction* in decision making performance. It appears that simplification sometimes led to the exclusion of information that was highly valued by participants, impeding performance: An accuracy measure assuming participants were responding purely on the basis of the most valued attributes explained the response data better than a measure assuming responses based on weighing multiple attributes against one another. Across conditions, simplification of information generally influenced both age groups similarly and was not particularly beneficial to older adults as initially hypothesized here, and elsewhere in the literature (e.g., Brand & Markowitsch, 2010).

Participants rated their political, medical and nutritional preferences across a range of attributes upon which later decisions were made. Our main analyses focused on a compensatory (Payne, 1976) accuracy measure that assumed decisions were made on the basis of weighing attributes against one another in order of their rated preferences. This mechanism of information integration has been hypothesized to be cognitively demanding (Payne, Bettman, & Johnson, 1993) and we initially expected to enhance performance by reducing the number of attributes that needed to be compared against one another. As discussed in the introduction, reducing such demands was expected to disproportionally benefit older adults (e.g., Bruine de Bruin, Parker, & Fischhoff, 2012) and this did occur in the nutritional condition where we saw age deficits for complex trials but not for simple trials. However, the political and medical conditions showed better performance for complex trials, where more attributes needed to be weighed against one another, than for simple trials.

Further analyses revealed that participants’ responses were better explained by a non-compensatory mechanism. When we defined the accuracy of a decision on the basis of its congruence with each participant’s most valued attribute (i.e., single-dimensional/non-compensatory decisions), this accuracy measure described the response data significantly better than the compensatory measure. Additionally, in contrast to Johnson (1990), there was no evidence that the use of compensatory mechanisms were more likely in young adults than in older adults: The Bayes factor provided ‘substantial’ evidence (Wetzels et al., 2011) in favor of the null hypothesis for the interaction between the compensatory and non-compensatory measures and age group (although this conclusion can only be applied to the particular measures that were used in this study). Additionally, when the analysis was conducted with just the single-dimensional/non-compensatory measure, age differences were nonsignificant and performance was higher for simple than for complex trials. Both young and older adults’ data was similarly explained better by the non-compensatory measure of accuracy which may have been driven by the simplicity of the tasks.

One limitation of the current study is the breadth of topics used as stimuli. As we initially hypothesized that complexity would have similar effects in all contexts, we had aimed to demonstrate this as widely as possible by covering the applied topics of politics nutrition and medicine. This meant that because the complexity manipulation may not have been the same in each condition, participants may have had more prior knowledge of one topic than another and this is a factor known to influence age differences (e.g., Badham et al., 2016). It may be necessary to move away from applied topics and to utilize abstract stimuli to evaluate more specific theoretical differences between young and older adults’ decision-making processes.

In the introduction, we argued that a potential limitation of earlier research investigating complexity manipulations with young and older adults was that simple trials often contained large amounts of information which may have still challenged older adults. The current study therefore used minimal amounts of information for assessment of decision making accuracy for simple trials. The single-dimensional measure of accuracy showed no significant age differences overall, indicating that the design was successfully simplified but it may be the case that the complex trials utilized were not complex enough to encourage compensatory processes and to challenge older adults. Nonetheless, there was little evidence of ceiling performance in the data and single-dimensional performance dropped as trials became more complex, demonstrating that complexity influenced difficulty. The interaction between accuracy measure and complexity also indicates that some participants may have adopted compensatory processing as trials became more complex (see also Table 2).

The applied nature of the study was partly utilized to aid simplification for older adults. As the decisions were based on participants’ real-life preferences there was no need for them to memorize experimental materials which may have disproportionately hindered older adults relative to young adults due to age-related memory deficits (Naveh-Benjamin & Ohta, 2012). By assessing preferences again at the end of each condition, it was possible to ascertain that age differences were not driven by forgetting the preferences. It has also been argued that real-world contexts help older adults engage with experimental materials and therefore provide a better assessment of their abilities (Peters & Bruine de Bruin, 2012). This may have been why age differences were not apparent in the final single dimensional analysis.

There is some research to suggest that individuals do not necessarily make decisions based on their stated preferences (Lindberg, Gärling, & Montgomery, 1989). To briefly test for this in the current data, the variance was compared between young and older adult’s chosen options for each complexity level and for each condition. This would establish if there were age differences in consistency of responding to *any* preference. There was no evidence of age differences in consistency for simple trials (all *BF*_10_ < 0.6) or for complex trials (all *BF*_10_ < 1.2) across the political, medical and nutritional conditions.

A consistent feature of complexity on age differences in the literature was in studies of information seeking, where older adults generally sought less information than young adults before making decisions (see, Mather, 2006, for a review and see the current introduction for examples). Mather hypothesised that age-deficits in executive function may lead to seeking less information as older adults are less able to deal with higher executive demands. However, this finding is also consistent with age deficits in self-initiated processing (cf., Craik & Byrd, 1982, where older adults perform better if given sufficent motivation) because older adults may be less motivated to continue seeking information. This could potentially explain why there is inconsistency of age deficits in the literature when the amount of available information is manipulated; just because the information is presented, it does not mean that older adults would have the motivation to utilise it. To account for this, future research may use manipulations of stimulus presentation to ensure that all available information is evaluated (e.g., forcing a response to each attribute before final evaluation - is this feature good or bad?). Another method to ensure compensatory processing could be to explicitly instruct participants to use all information presented; results from the memory literature have shown that older adults can utilise optimal strategies if encouraged to do so (e.g., Naveh-Benjamin, Brav, & Levy, 2007) and this may equally apply to decision making.

Individual differences have also been shown to influence strategy use in decision making. Shiloh, Koren, and Zakay (2001) showed links between compensatory decision-making style and the subjective complexity of the decision. They argued that the perceived difficulty of a task may prevent decision making or lead to non-optimal decisions. This is a pertinent problem for older adults who often have subjective cognitive complaints which are not strongly related to their actual cognitive performance (see Burmester, Leathem, & Merrick, 2016, for a review) as they may begin to utilise simpler, and non-optimal decision-making processes. This may be further problematic for old-old adults who typically have greater subjective cognitive concerns (than older adults) that are also not linked to actual cognitive performance (Pearman, Hertzog, & Gerstorf, 2014; Shmotkin, et al., 2013). Future ageing research on decision making should aim to include a subjective difficulty measure to account for age differences in cognitive confidence, especially if aiming to assess compensatory processing.

Overall, the current data raises important issues relevant to the understanding of decision making. In contrast to prior theory suggesting that simplifying important decision-making information would be particularly beneficial to older adults (Peters & Bruine de Bruin, 2012), the current results indicate that the process of simplification via exclusion of information can hinder decision performance for all age groups. Our data shows that young and older adults’ decisions were explained well by a measure that assumed participants responded purely based on the single piece of information they valued most. Therefore, simplification that excludes this information could be particularly detrimental to performance. We recommend that professionals preparing information materials for older adults should be fully informed of the recipients’ preferences and priorities before simplification via exclusion and should seek other methods of simplification where possible such as improved organization of material about which decisions are to be made (cf. Zaval et al., 2015).
